# *Popowia bachmaensis* (Annonaceae), a new species from Bach Ma National Park, Central Vietnam

**DOI:** 10.3897/phytokeys.65.8792

**Published:** 2016-07-12

**Authors:** Nguyen Van Ngoc, Shuichiro Tagane, Hoang Thi Binh, Hironori Toyama, Norikazu Okabe, Chinh Nguyen Duy, Tetsukazu Yahara

**Affiliations:** 1 Center for Asian Conservation Ecology, Kyushu University, 744 Motooka, Fukuoka, 819-0395, Japan; 2 Department of Biology, Dalat University, 01 – Phu Dong Thien Vuong, Dalat, Vietnam

**Keywords:** Annonaceae, Bach Ma National Park, new species, Popowia, Vietnam

## Abstract

A new species, *Popowia bachmaensis* Ngoc, Tagane & Yahara, **sp. nov.** is described from Bach Ma National Park in Thua Thien Hue Province, Central Vietnam. This species is morphologically similar to *Popowia pisocarpa* (Blume) Endl. ex Walp., but can be readily distinguished from it by its lower stems, smaller leaves, shorter flowering pedicels, shorter carpels, longer sepals and inner petals. A detailed description, comprising illustrations, and supplemented with DNA barcodes of the two regions of *rbcL* and *matK*, are provided.

## Introduction

*Popowia* Endlicher is a small genus of the family Annonaceae. It was firstly described in the Genera Plantarum secundum Ordines Naturales for the type species, *Popowia pisocarpa* (Blume) Endl. ex Walp (Endlicher 1839). The species is a shrub or a small tree, characterized mainly by its (sub)granular leaves with asymmetric base, inner petals being larger than outer ones, apically broadly flat-topped to slightly concave stamen connectives, 1 or 2(–4) ovule(s) per carpel (Moeljono 2009; Li and Gilbert 2011). The genus comprises approximately 50–90 species, but many of these could in fact belong to other genera (Sinclair 1955). The majority are distributed in tropical Africa, and also recorded from Madagascar, India, Myanmar, Thailand, Malaysia, Vietnam, China, Indonesia, Philippines, and Papua New Guinea to Australia. (Sinclair 1955; Moeljono 2009; Li and Gilbert 2011).

The genus *Popowia* has been classified within tribe Mitrephoreae Hook. f. & Thomson with the genus *Goniothalamus* (Blume) Hook.f. & Thomson, *Mitrephora* Hook.f. & Thomson, *Neouvaria* Airy Shaw, *Oxymitra* Hook. f. & Thomson and *Pseuduvaria* Miq. (Sinclair 1955; Moeljono 2009). Recent molecular analyses suggested its placement under tribe Miliuseae including a total of 25 genera and ca. 510 species and also strongly supports its monophyly with *Polyalthia* s. str. as a sister clade (Chatrou et al. 2012; Xue et al. 2012; Mols and Keβler 2013; Chaowasku et al. 2014). The species of *Popowia* and *Polyalthia* s. str. are usually characterized by the asymmetrical leaf base, but they are differentiated in the patterns of secondary leaf venation (eucamptodromous in *Popowia* vs. brochidodromous in *Polyalthia* s. str.), and the number of ovules per carpel [1 or 2(–4) vs. 2–6] (Xue et al. 2012).

In a recent taxonomic revision of Annonaceae in Vietnam, Bân (2000) reported three species with one variety of *Popowia*: *Popowia cambodica* Finet & Gagnep., *Popowia cambodica var. canaensis* Bân, *Popowia helferi* Hook.f. & Thomson and *Popowia pisocarpa*. However, specimens of “*Popowia cambodica* and Popowia cambodica var. canaensis” identified by Bân (2000) are identical with *Polyalthia debilis* (Pierre) Finet & Gagnep (digitalized specimen images examined). As for “*Popowia helferi*”, the description by Bân (2000) does not match with the original description by Hook and Thomson (1872). Therefore, only *Popowia pisocarpa* can be considered as a reliable record in Vietnam.

Here, the second Vietnamese species of *Popowia* is reported, which was found in Bach Ma National Park, Phu Loc District, Thua Thien Hue Province. This national park was established in 1991 with a total area of 37,487 hectares and is recognized as a biodiversity hotspot because of its unique topography, high species richness and highly threatened biodiversity. The peak of Bach Ma Mt. is 1,450 m high and is covered by clouds almost throughout the year. The park preserves virgin forests, which depending on their altitudinal distribution can be classified as follows: seasonal evergreen forests, hill evergreen forests, and lower montane forests. So far 2,373 species of vascular plants, accounting for approximately 17% species of the total flora of Vietnam, have been recorded from the National Park (Bach Ma National Park 2016).

During our botanical inventory of Bach Ma National Park in 2015, a new species of genus *Popowia* was discovered, *Popowia bachmaensis* Ngoc, Tagane & Yahara, sp. nov. Here, it is described, illustrated and the DNA barcodes are provided of the two plastid regions *rbcL* and *matK* (CBOL Plant Working Group 2009) of the new species.

## Material and methods

### Morphological analysis

The new species was recognized through literature review, examined specimens in the herbaria ANDA, BKF, BM, BO, HN, K, L, P and online digitized plant specimens (e.g. JSTOR Global Plants). The measurements of sepals, petals, carpels and stamens was measured using a digital caliper (Absolute Digimatic 547-401, Mitutoyo, Japan, resolution 0.001 mm).

### DNA barcoding

Leaf pieces were dried using silica-gel in the field and DNA isolation was performed by the CTAB method (Doyle and Doyle 1987) with minor modifications described in Toyama et al. (2015). Two DNA barcode regions were amplified and sequenced according to published protocols (Kress et al. 2009, Dunning and Savolainen 2010).

## Taxonomy

### Popowia bachmaensis

Taxon classificationPlantaeMagnolialesAnnonaceae

Ngoc, Tagane & Yahara sp. nov.

urn:lsid:ipni.org:names:77155959-1

Figs 1 , 2

#### Diagnosis.

Similar to *Popowia pisocarpa* (Blume) Endl. ex Walp., but distinguished from that species by having small habits (30–60 cm tall vs. 3–7 m tall in *Popowia pisocarpa*), smaller leaves (4.6–10.8 cm × 2.0–5.6 cm vs. 5.5–14 cm × 2.5–7 cm in *Popowia pisocarpa*), longer petioles (ca. 1.8–3 mm vs. 2–5 mm long) shorter flowering pedicels (2–3.5 vs. 4–7 mm long), longer sepals (ca. 3 mm vs. 2 mm long), longer inner petals (6 mm vs. 3 mm long), shorter carpels (2.1 mm vs. 10 mm long) (The measurements of *Popowia pisocarpa* derive from Sinclair 1955).

**Figure 1.**
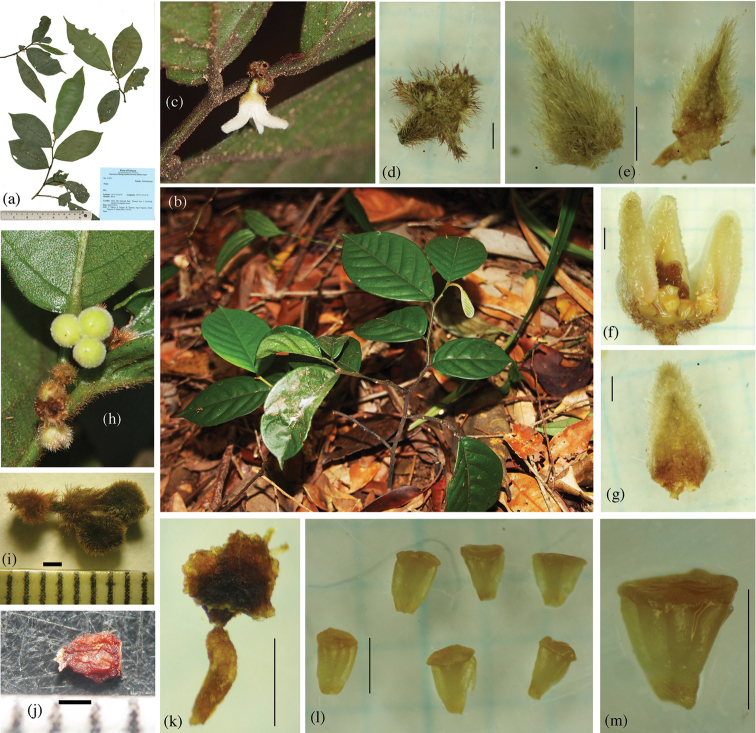
*Popowia bachmaensis*, sp. nov. **a** Holotype (KYO) **b** habit **c** flower **d** pedicel and sepals **e** sepals **f** section of flower **g** inner petal **h** fruit on branch **i** dried fruit **j** seed **k** carpel **l, m** stamens. Materials from *Yahara et al. V2557* (KYO). Scale bars: **d–g, i–m** =1 mm.

**Figure 2.**
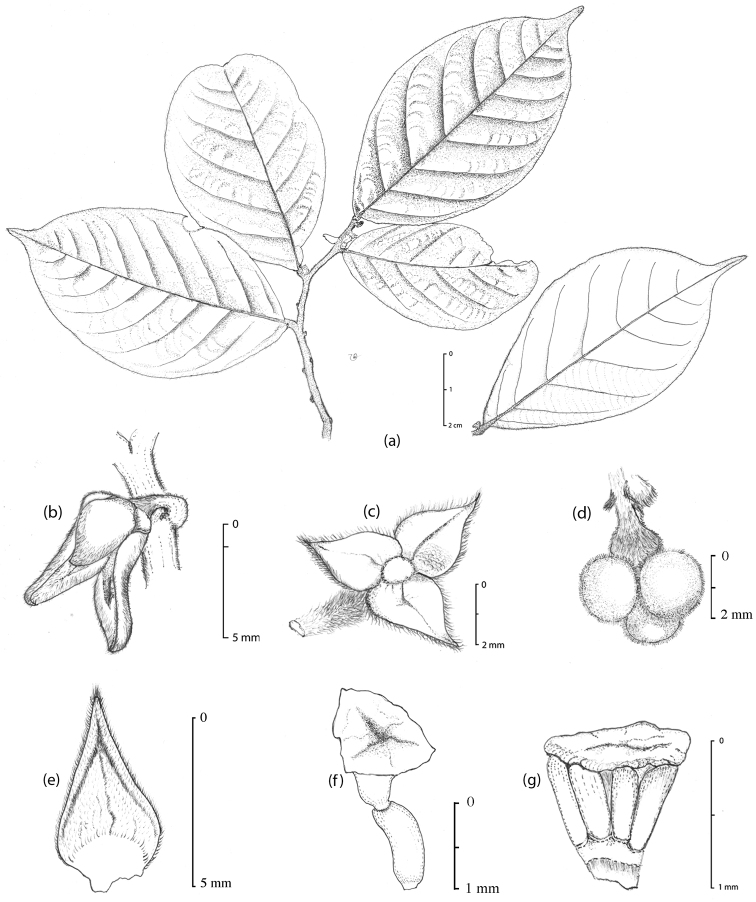
Illustration of *Popowia bachmaensis*, sp. nov. **a** Leafy twig **b** Flower **c** Pedicel and sepals **d** Fruit **e** Inner petal **f** Carpel and **g** Stamen. Materials all from Yahara et al. *V2557* (FU). Drawn by Ngoc & Binh.

#### Type.

VIETNAM. Thua Thien Hue Province, Bach Ma National Park, in evergreen forest, 16°13'41.60"N, 107°51'09.35"E (DMS), alt. 485 m, 23 May 2015, with flowers and young fruits, *Yahara T*, *Tagane S.*, *Toyama H.*, *Nguyen Ngoc*, *Nguyen Chinh*, *Okabe N. V2557* (holotype: KYO!; isotypes: BKF!, DLU!, FU!, the herbarium of Bach Ma National Park!).

#### Description.

Shrubs, 30–60 cm tall. Young twigs hirtellous with yellowish brown hairs, glabrescent, blackish. Leaves alternate; petioles 1.8–3 mm long, hirtellous; blades elliptic, elliptic-obovate, obovate, (3.7–)4.6–10.8(–15) × 2.0–5.6 cm, length/width ratio 1.7–2.5(–3.2), base obtuse, usually asymmetric, apex acuminate, acumen up to 1.4 cm long, margin entire, ciliate, papery, dull greyish green to dull blackish brown adaxially, pale green, dull greyish green, or greyish brown abaxially, minutely granular, pubescent on both surfaces when young, glabrescent adaxially when old; midribs prominent abaxially, pubescent on both surfaces; secondary veins 7–11 pairs, arising at angle of 45–55 degrees from a midrib, prominent abaxially when dry, pubescent on both surfaces; tertiary veins faintly visible, scalariform-reticulate. Inflorescences extra-axillary or leave-opposed, fascicles of 1–3 flowers. Pedicels 2–3.5 mm long, hirtellous; bracts triangular, ca. 1 mm long, ca. 0.5 mm wide, brownish pubescent outside and margin, glabrous inside, bracteoles caducous. Sepals 3, broadly ovate, ca. 3 × 3 mm, pubescent outside, glabrous inside. Petals 6, white; outer petals ovate-triangular, ca. 2.7 × 1.5 mm, pubescent outside, glabrous inside; inner petals narrowly ovate-triangular, ca. 6 × 3.2 mm, pubescent on both surfaces except lower part of inside. Stamens 22 per flower, reverse truncated pyramid, ca. 1.1 × 0.8 mm, glabrous, the connective truncate, flat-topped or slightly concave, ca. 0.1–0.2 mm long; anthers ca. 0.7 mm long. Carpels 6 per flower, ca. 2.1 mm long; ovary ca. 1.1 mm long, glabrous; stigmas and pseudostyles reverse conical, ca. 1 × 0.7 mm. Immature fruiting pedicels ca. 3.5–4.0 mm long, pubescent with reddish brown hairs; monocarp three, globose, ca. 2.2 mm in diam., pubescent with short white hairs, hairs blackish brown when dried. Seeds one per monocarp, ca. 1.5 mm long, reddish brown, glabrous, furrowed when dried.

#### Distribution and habitat.

Vietnam (so far known only from its type locality).

#### Phenology.

Mature flowers and young fruits were collected in May.

#### Etymology.

The specific epithet ‘*bachmaensis*’ is derived from the type locality, Mt. Bach Ma, Vietnam.

#### GeneBank accession No.

*Yahara et al.* V2557: LC090861 (*rbcL*), LC090860 (*matK*).

#### Conservation status.


 Data Deficient (DD). During the botanical inventory carried out from 21 to 28 May 2015 from the foot to the top of Mt. Bach Ma, 15 individuals of *Popowia bachmaensis* were observed in all. They were found on the slopes of secondary hilly evergreen forest, at ca. 500 m elevation. Among them, only two individuals produced flowers, one produced fruits, and the others are just saplings. According to the population size observed, this species can be qualified as Critically Endangered (CR) (IUCN 2012). However, only a limited area of the forest in the vicinity of the type locality was surveyed, and further field surveys are needed to determine the actual population size within Bach Ma National Park. The forest of the type locality was slightly disturbed in the past, but it is now well-protected from human activities.

## Supplementary Material

XML Treatment for Popowia bachmaensis
